# Advances in Diagnosis and Therapeutics in Recurrent Autoimmune Pericarditis in Pregnancy

**DOI:** 10.1016/j.jaccas.2023.102046

**Published:** 2023-12-06

**Authors:** Samia Mazumder, Rehan Karmali, Parvathy Sankar, Muhammad Majid, Alveena Batool Syed, Felix Berglund, Faiz Ahmed, Jameson Mitchell, Brittany Weber, Allan L. Klein

**Affiliations:** a Department of Internal Medicine, Cleveland Clinic, Cleveland, Ohio, USA; b Cleveland Clinic Lerner College of Medicine, Cleveland, Ohio, USA; c Division of Cardiovascular Medicine, Department of Medicine, Brigham and Women’s Hospital, Harvard Medical School, Boston, Massachusetts, USA; d Center for the Diagnosis and Treatment of Pericardial Diseases, Section of Cardiovascular Imaging, Department of Cardiovascular Medicine, Heart, Vascular and Thoracic Institute, Cleveland Clinic, Cleveland, Ohio, USA

**Keywords:** autoimmune disease, pericarditis, pregnancy, recurrent pericarditis, systemic lupus erythematosus

## Abstract

Pericarditis in pregnancy is uncommon, and there is a paucity of data regarding the safety and efficacy of conventional therapy. We describe a complex case of recurrent pericarditis in the setting of pregnancy and newly diagnosed systemic lupus erythematosus and discuss the challenges in managing this subset of patients.

## Central Illustration

**Figure:**
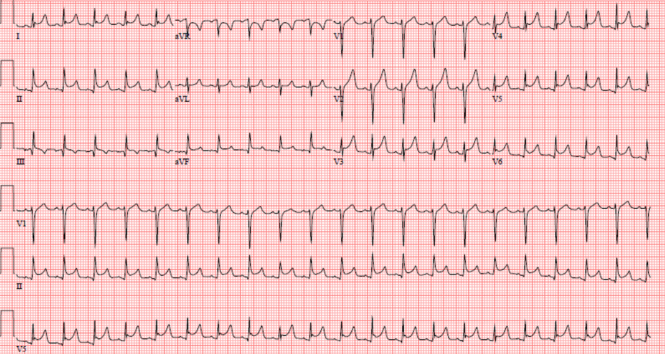

## History of Presentation

A 28-year-old woman at 24 weeks gestation presented with sudden-onset vision loss,; bilateral leg swelling; and new, nonspecific joint pain. She was found to have bilateral optic neuritis of unclear etiology and was subsequently treated with a course of high-dose steroids and plasmapheresis. While hospitalized, she complained of sharp pleuritic and positional chest pain that was not relieved by conservative measures, including acetaminophen and topical analgesia. The chest pain worsened with inspiration and lying flat and improved when leaning forward. Two days later, she continued to endorse persistent, pleuritic, nonradiating chest pain. Her vital signs were significant for hypotension (blood pressure: 97/62 mm Hg) with distant heart sounds, which was a marked change from her normal vital signs on presentation.

**Learning Objectives**

• To understand the management of autoimmune pericarditis.
• To understand key differences in management in patients who are also pregnant.
• To emphasize the importance of a multidisciplinary team with a cardiologist, rheumatologist, and obstetrician when delivering care.

## Past Medical History

Past medical history includes obesity (body mass index: 31.2 kg/m^2^) and iron-deficiency anemia. There was no known history of autoimmune disease or malignancy. She also denied recent travel, new medications and supplements, or new exposures to radiation or toxins.

## Differential Diagnosis

Differential diagnoses include myocardial infarction, spontaneous coronary artery dissection, viral pericarditis, cardiac tamponade, costochondritis, malignancy, and autoimmune disease.

### Investigations

**Figure 1:**
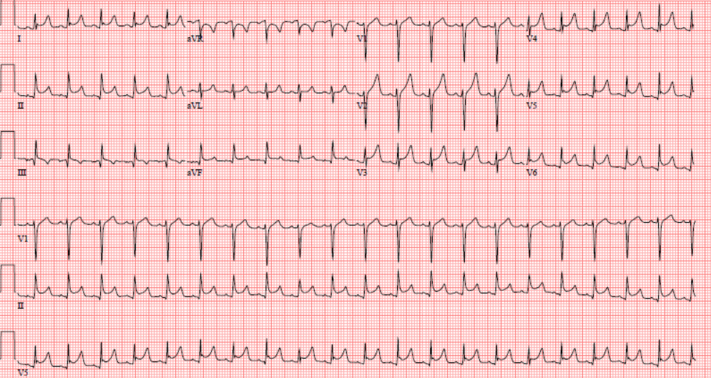
Diffuse ST-Segment Elevation and PR-Segment Depression Highlighted in Multiple Leads

**Figure 2:**
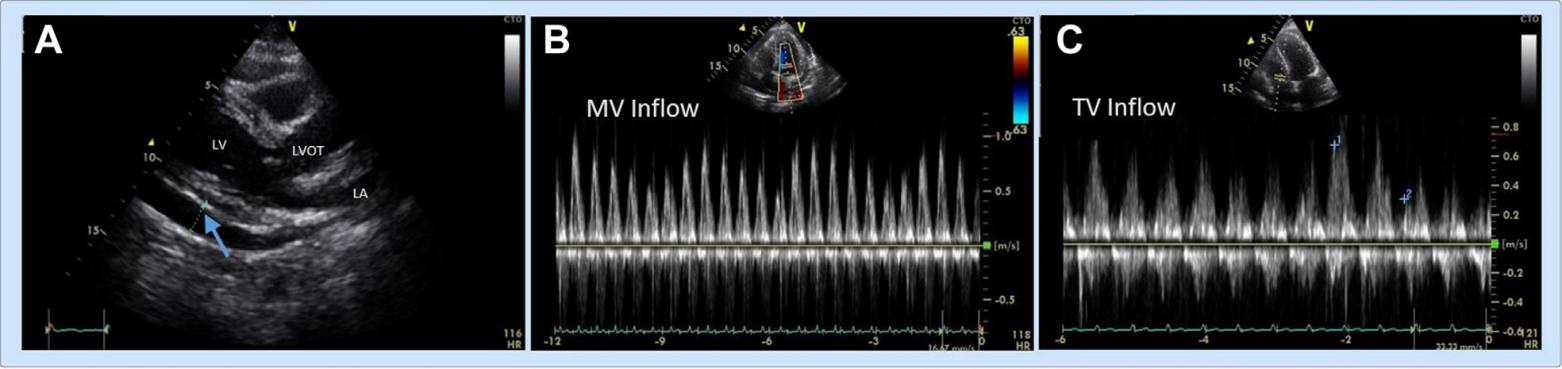
Echocardiographic Findings (A) Echocardiogram on initial presentation. A large pericardial effusion is seen on the long-axis view (blue arrow), left ventricle (LV), left atrium (LA) and left ventricle outflow tract (LVOT) shown. (B, C) Respiratory variation across the mitral valve (MV) and tricuspid valve (TV). The mitral valve peak percentage difference is 48.4%, and the tricuspid valve peak percentage difference is –119.4%.

She was initially followed with serial high-sensitivity troponin tests, the results of which were persistently negative and suggested lack of myocardial involvement. Electrocardiograms (ECGs) showed diffuse ST-segment elevation with PR depression (Figure 1). Inflammatory markers were significantly elevated; erythrocyte sedimentation rate was 36 mm/h, and C-reactive protein trended up from 1.1 to 3.3 mg/dL. Urine analysis was significant for few red blood cells, but no casts. Transthoracic echocardiogram (TTE) showed a large circumferential pericardial effusion with evidence of early diastolic right ventricle collapse, suggestive of increased intrapericardial pressures (Figure 2A, Video 1). There was respiratory variation across the mitral and tricuspid valves: 26% across the mitral valve and 58% across the tricuspid valve (Figures 2B and 2C). The IVC was mildly dilated (2.2 cm) and collapsed <50% with inspiration (Video 2). The patient was in cardiac tamponade, and she underwent an emergent pericardiocentesis, which removed approximately 300 mL of serosanguinous fluid. Pericardial fluid cytology was sent, and the result was negative for any evidence of malignancy or bacterial infections such as tuberculosis.

In addition, an autoimmune panel was ordered to assess for an underlying autoimmune disease. Anti-nuclear antibody (ANA), anti–double-stranded DNA antibody (via enzyme-linked immunosorbent assay), anti-neutrophilic cytoplasmic antibodies, anti-Ro (Anti-Sjogren's Syndrome A), anti-La (Anti-Sjogren's Syndrome B), anti-smith, and extractable nuclear antigen test results were negative. Complement levels, C3 and C4, were also assessed, and they were within normal limits.

The patient had elevated cardiolipin immunoglobulin M antibodies (14 MPL) during her initial presentation. Additionally, she underwent a kidney biopsy, which showed mild immune-complex–mediated focal mesangial proliferative glomerulonephritis. Per the pathologist review, the findings were likely related to an underlying autoimmune process. The constellation of symptoms, including polyarthritis, optic neuritis, pericarditis, and glomerulonephritis in the setting of elevated cardiolipin immunoglobulin M antibody levels led to a diagnosis of systemic lupus erythematosus (SLE) based on the Systemic Lupus Collaborating Clinics classification. The Systemic Lupus Collaborating Clinics classification requires satisfying 4 total criteria, including at least 1 clinical and 1 immunologic criterion.^1^ Although SLE typically has a high positive rate of ANA, ANA-negative SLE has been reported in the literature with rates as high as 6.2% in newly diagnosed SLE.^2^ We strongly suspect that the patient described in this case had an ANA-negative SLE.

## Management

Because of the patient’s hemodynamic instability from tamponade physiology seen on TTE, she was admitted to the cardiac intensive care unit and underwent pericardiocentesis and pericardial window. To treat her pericarditis and concomitant SLE, she was initially started on prednisone 60 mg. Because of the lack of clarity in treatment guidelines, her pregnancy posed a therapeutic challenge.

A multidisciplinary team consisting of her cardiologist, rheumatologist, and obstetrician held an extensive risk-benefit discussion with the patient regarding potential treatment options. The patient was advised that azathioprine carried a risk of low fetal birth weight and prematurity and low risk of teratogenicity and fetal malformations. Although colchicine was not strictly contraindicated in pregnancy, the data were scarce in terms of the risk of teratogenicity and fetal malformations. Because of her diagnosis of Focal segmental glomerulosclerosis and the potential for renal damage, the patient opted for low-dose colchicine 0.3 mg twice a day and azathioprine 50 mg twice a day.

Following pregnancy, prednisone was gradually tapered off, hydroxychloroquine 200 mg daily was added, and her dose of colchicine was increased to 0.6 mg twice a day. This treatment regimen was carefully selected after weighing the risks and benefits and determining the best course of therapy to treat and prevent autoimmune pericarditis recurrence in the setting of pregnancy.

**Figure 3:**
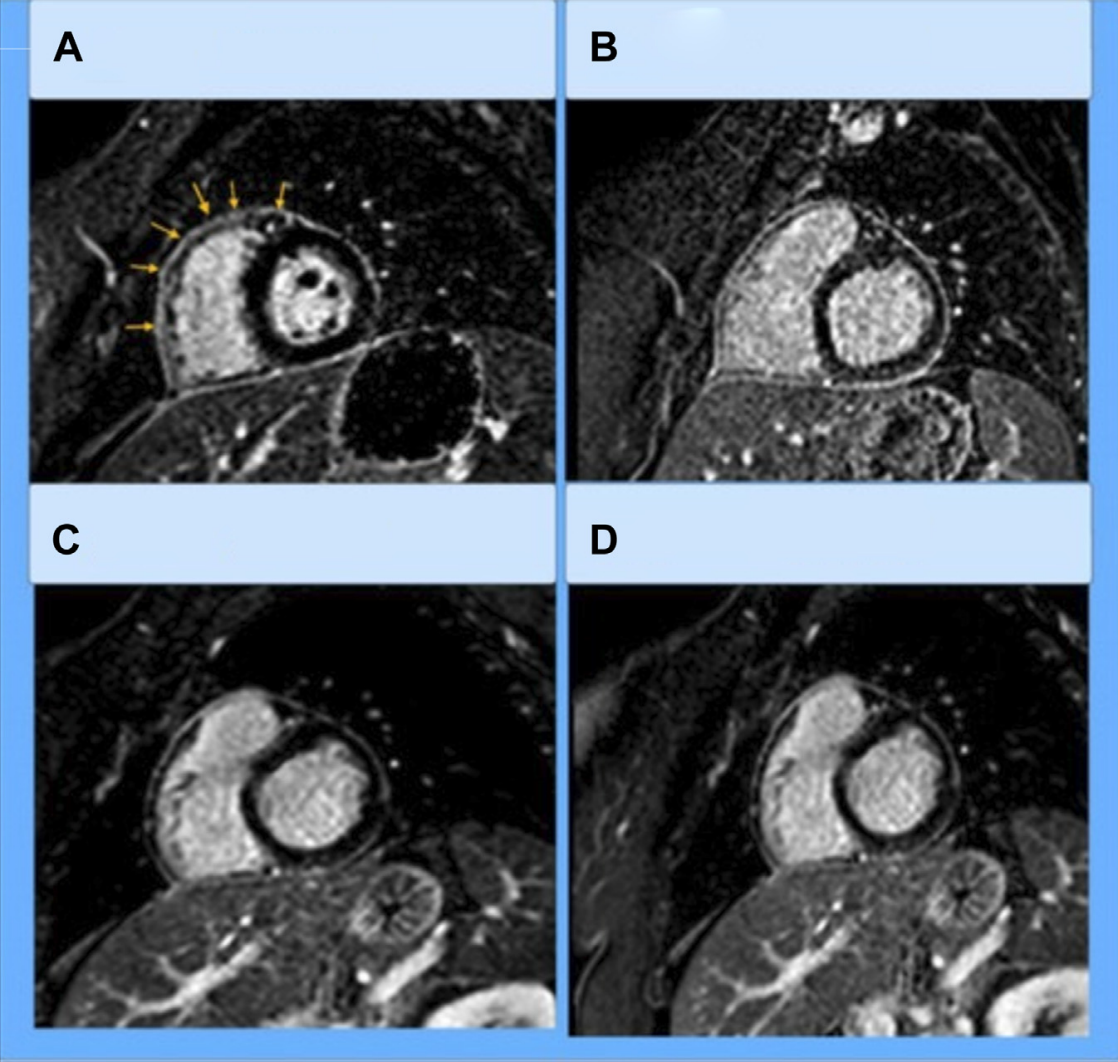
Timeline of Cardiac Magnetic Resonance Showing Progression of Pericardial Disease (A) Moderate circumferential pericardial enhancement 1 month after presentation (yellow). (B) The interval decrease 5 months after presentation. (C) Only subtle delayed enhancement 19 months after presentation. (D) Stable mild delayed enhancement 43 months after presentation.

Serial follow-up included inflammatory markers, ECGs, echocardiogram, and cardiac magnetic resonance (Figure 3). Although pericarditis in the setting of pregnancy challenged current therapeutic guidelines, her treatment plan resulted in a healthy delivery and controlled, chronic pericarditis on yearly follow-up.

## Discussion

The etiology of pericarditis is variable and may often be attributable to idiopathic causes have bacterial, viral, or autoimmune origin. Echocardiogram is the mainstay of diagnosis and is supported by clinical presentation, ECG changes, and physical examination findings (pericardial friction rub). Most cases of pericarditis are managed medically without the use of invasive therapies, such as a pericardial window.^3^ Chronic, recurrent pericarditis creates an additional therapeutic challenge that can be further complicated by pregnancy and undiagnosed autoimmune disease. Pregnancy complicates the treatment of pericarditis because many of the conventional therapies such as (nonsteroidal anti-inflammatory drugs [NSAIDs] and colchicine) are either contraindicated or do not have evidence supporting their use.^3^

There are no formal guidelines that specify pericarditis management in pregnancy. However, there have been several case series and case studies that have discussed management and have contributed to the creation of guiding principles when approaching these patients. The European Society of Cardiology has commented on the use of certain therapies during pregnancy to manage recurrent pericarditis. Per their findings, NSAIDs, colchicine, corticosteroids, Imuran (Azathioprine), and intravenous immunoglobulin are relatively safe in the first, second, and third trimesters (Table 1). We used a multimodality image-based approach with repeat cardiac magnetic resonance to follow the course of her recurrent pericarditis in conjunction with following the existing guiding principles.

**Table 1:** Summary of Current Therapies for Pericarditis in Pregnancy

	First Trimester	Second Trimester	Third Trimester	Lactation
NSAIDs	Allowed	Allowed	Avoid	Allowed
Colchicine	Allowed	Allowed	Allowed	Allowed
Corticosteroids (preferred dose: prednisone <20 mg/d)	Allowed	Allowed	Allowed	Allowed
Biologics	Avoid	Avoid	Avoid	Allowed
DMARDs (methotrexate, mycophenolate mofetil)	Avoid	Avoid	Avoid	Avoid

The table is organized by therapies approved by trimester and throughout lactation.

DMARD = disease-modifying antirheumatic drug; NSAID = nonsteroidal anti-inflammatory drug.

The treatment of autoimmune pericarditis incorporates many components of standard pericarditis treatment (NSAIDs, colchicine, steroids); however, it also favors the use of biologics such as interleukin 1 receptor antagonists and disease-modifying antirheumatic drugs, such as methotrexate and mycophenolate mofetil. Biologics such as interleukin-1 receptor antagonists, methotrexate, and mycophenolate mofetil are not recommended for use during any point while a patient is pregnant because they may cause adverse reactions to the fetus.^4^

Although NSAIDs continue to remain the standard of treatment during the first and second trimesters, they should be avoided in third trimester to prevent premature closure of patent ductus arteriosus.^5^ There is no definitive consensus on whether NSAIDs are safe before conception. Some data point toward them affecting ovulation and increasing chances of miscarriage. Ibuprofen is preferred because of its better safety profile in terms of cross-placental transfer.^5^^,^^6^

Colchicine and low-dose steroids (0.2-0.5 mg/kg prednisone) play a central role in the treatment of recurrent pericarditis. Colchicine blocks mitotic division in metaphase but has been deemed safe during conception and pregnancy. Most of the safety data for colchicine are derived from a meta-analysis of 554 pregnancies where colchicine was continued for familial Mediterranean fever with no congenital fetal defects noted.^7^ Steroids are also safe during conception and pregnancy, with ideal agents being prednisone or prednisolone. Rapid metabolism of steroids and dosing of <20 mg/day limits fetal exposure.^8^

There is a lack of data on the safety of azathioprine on pericarditis. It is a purine metabolism antagonist. The placenta metabolizes azathioprine to an inactive metabolite, which has been detected in fetal blood. It has not shown to have any teratogenic effects, but higher rates of other pregnancy complications, including low birth weight, prematurity, and jaundice, have been reported.^8^ The mentioned pharmacologic agents are all safe during breastfeeding. Methotrexate and mycophenolate mofetil are the 2 agents contraindicated during conception, pregnancy, and breastfeeding.^8^ Outcomes of pregnancy in patients with pericarditis are good when the patients are managed by a multidisciplinary team, which has been evident in cohort analysis with pregnant patients who have a history of recurrent pericarditis.^9^

A multidisciplinary approach to treating pericarditis was paramount in ensuring a positive maternal-fetal outcome. By having an open lines of communication among multiple specialties, all aspects of this case were weighed carefully before making a decision on the best course of therapy. This case highlights the importance of multidisciplinary care when treating a patient with a complex condition with several variables that may affect her treatment outcomes.

## Follow-Up

The patient described in our case had a total of 4 children with 3 pregnancies while being on colchicine 0.6 mg twice daily and azathioprine 150 mg at different timepoints. Following initial therapy, she did not have any documented evidence of hospital admissions or emergency department visits for symptomatic pericarditis with subsequent pregnancies, and her condition remained well controlled on the current regimen. All pregnancies were full-term vaginal deliveries. Newborn health assessment via Apgar scoring ranged between 7 and 9 for all 4 children, indicating normal births. There was no documented history of prematurity or evidence of fetal malformations or organ injury in any of her children. Her most recent regimen includes colchicine 0.6 mg twice daily and hydroxychloroquine 200 mg. Her subsequent imaging results have all been negative for pericardial effusion.

## Conclusions

This case supports the use of colchicine for presumed autoimmune pericarditis and highlights the importance of shared decision making between physicians and patients when choosing azathioprine for treatment. These patients pose the most challenges with the initial diagnosis and management choice. However, a multidisciplinary team approach with the trial of the discussed agents gave this patient successful pregnancy outcomes.

## Funding Support and Author Disclosures

The authors have reported that they have no relationships relevant to the contents of this paper to disclose.

## Appendix

Video 1 Parasternal Long-Axis View of Initial TTE Showing Tamponade Physiology

Video 2 Short-Axis View of Initial TTE Showing Tamponade Physiology
